# Empirical analysis of the impact of the digital economy on the green transformation of manufacturing: Evidence from China

**DOI:** 10.1371/journal.pone.0289968

**Published:** 2023-08-23

**Authors:** Chunjie Jia, Meng Shang, Junwei Cao, Yu Liu

**Affiliations:** 1 School of Flight, Anyang Institute of Technology, Anyang, China; 2 School of Business, Yangzhou University, Yangzhou, China; University of Palermo: Universita degli Studi di Palermo, ITALY

## Abstract

In this study, the entropy method and the Super-SBM model with unexpected output are used at first to calculate the digital economy development index and the level of green transformation in manufacturing. Then, a range of multi-dimensional empirical methods, including fixed effects models, threshold models, and mediation models, are applied to analyze the characteristics shown by the impact of digital economy development on the green transformation of manufacturing. The research results are obtained as follows. Firstly, the digital economy contributes significantly to promoting the green transformation of manufacturing after excluding the macro-system environmental effects, conducting such robustness tests as stepwise regression and introducing instrumental variables. Secondly, there is a nonlinear relationship between the development of the digital economy and the green transformation of manufacturing with an increasing marginal effect. Lastly, it is revealed through mechanism analysis that the digital economy promotes the green transformation of manufacturing by enhancing the capabilities of green technological innovation and rationalizing industrial upgrading, with the partial mediation effects reaching 21.2% and 21.8%, respectively. Despite the contribution of digital economy to the advanced upgrading of industries, there is no mediation effect exhibited. In addition to confirming the path of achieving the green transformation of manufacturing through the digital economy, these results also guide the government on how policies can be formulated and improved to grow the digital economy and promote the green transformation of manufacturing.

## Introduction

In recent decades, the increase in carbon dioxide emissions has caused a profound impact on the global climate, which leads to the exacerbation of global warming [1]. According to the data published by the International Energy Agency (IEA), global carbon emissions amounted to 35 billion tons in 2020, with industrial manufacturing emissions accounting for nearly a quarter, or 9 billion tons. Allowing for a significant contribution of the industrial manufacturing carbon emissions to global emissions [2], to promote the green transformation of manufacturing is considered essential for improving environmental quality and enhancing energy efficiency.

Currently, green transformation has been recognized as a new driving force for global economic restructuring and environmental governance, with various incentive measures implemented by the governments around the world to promote the development of green transformation [3]. For example, the Chinese government proposed the "dual-carbon" goal at the United Nations General Assembly in 2020, striving to peak carbon dioxide emissions before 2030 and achieve carbon neutrality by 2060. As the central pillar of economic development, manufacturing lays a foundation for the high-quality growth of national economy. In 2020, the total energy consumption by the manufacturing industry in China reached 27.9 billion tons of standard coal, accounting for 56.1% of the national total. However, in terms of carbon emissions, the CO_2_ emissions from the manufacturing industry reached 6.946 billion tons in 2020, accounting for 70.2% of the national total, which has a serious effect on sustainable economic development. It is imperative to accelerate the green transformation and upgrading of the manufacturing industry as the development of the manufacturing industry is subjected to the dual pressures of foreign competition and strict environmental protection requirements [4].

The digital economy represents an emerging form of economy arising from the development of new information technologies and the Internet. At present, it has provided an important driving force needed to promote the improvement of economic quality, accelerate power upgrading, and facilitate efficiency transformation, because it overcomes the resource endowment constraints and diminishing returns to production factors encountered by traditional production factors [5]. According to the 2022 Global Digital Economy Report, the global digital economy has reached a scale of 38.1 trillion yuan, which is an increase by 5.1% year on year. The digital economy has demonstrated a strong resilience through a steady growth achieved against the trend [6]. It represents the future trend to transform traditional manufacturing into digital and intelligent manufacturing, showing the characteristics of digitization such as the reform of production methods driven by big data and complex manufacturing systems, the networked circulation of production elements, and the construction of the entire value chain [7]. Undeniably, the digital economy provides new options for the green transformation and upgrading of manufacturing through the in-depth integration of digital technology and physical enterprises, the innovation breakthroughs of new digital technologies such as big data, artificial intelligence, and the Internet of Things, as well as the effective interaction between manufacturing and service industries [8]. On the one hand, the growth of the digital economy enhances the social productivity of the manufacturing industry by promoting the application of big data and information technology and industrial intelligent equipment in technology development, production and processing, and business management [9]. On the other hand, digital technology is effective not only in reducing the costs of traditional industry operation and maintenance but also in managing energy production and consumption, which is conducive to the green transformation of manufacturing. From the above analysis, it can be found out that the growth of digital economy plays an important role in promoting the green transformation of manufacturing. So measuring the current level of green transformation in the manufacturing industry is the basis for subsequent research.

Non-parametric methods are used by most researchers to measure the efficiency of green transformation in the context of manufacturing. In the study of Li Xin’an and Li Hui [10], the inputs include such internal input variables as funding, personnel, R&D expenditures, and total energy consumption, while the outputs include such total output variables as manufacturing output, GDP, and the sales revenue of new products. The efficiency of green development was measured by using the Super-SBM model, with industrial wastewater discharge as the unexpected output. By taking carbon dioxide as the unexpected output, Emrouznejad and Yang [11] evaluated the Chinese manufacturing for its ecological and environmental efficiency. Liu Xinzhi and Kong Fangxia [12] adopted the Super-SBM model to calculate the level of green transformation in the context of Chinese urban industry. To sum up, these studies have provided valuable references for analyzing the efficiency of green transformation in manufacturing. In this article, unexpected outputs are factored into the framework of efficiency measurement, which is based on the study of Timmer and Szirmai [13]. The Super-SBM model is applied to measure the efficiency of green transformation in provincial-level manufacturing across China.

Undoubtedly, the development of the digital economy plays a vital role in the green transformation of manufacturing. So far, scholars have taken different perspectives to explore the relationship between the digital economy and green transformation of manufacturing. The major influencing factors of green transformation can be categorized into internal and external ones. Internal factors include technological innovation, labor level, energy structure, informationization level, financing capacity, etc. [14], while external factors include environmental regulations, resource endowments, government behavior, market competition environment, etc. From the perspective of governments, enterprises can be further induced and forced to embrace the green transformation path through a range of policies such as environmental regulations and green transformation subsidies [15]. As indicated by Kutin et al. [16], the transformation and upgrading of the manufacturing industry can be achieved through technology introduction, absorption, and integration in the process of integrating digital technology and manufacturing, which is conducive to improving the overall efficiency of the industry chain. Al-Okaily et al [17] used structural equations to validate the effectiveness of data warehouse technology in digital transformation of enterprises using the case of Jordanian banks. Tsolakis et al [18] explored the joint implementation of artificial intelligence (AI) and blockchain technology (BCT) in tuna supply chain and found that digitalization can transform the operational performance of end-to-end supply chain. Chatterjee et al. [19] found that digitization with the help of Industry 4.0 technologies will help restaurant companies to quickly overcome the limitations experienced since the end of the COVID-19 outbreak.

Although the positive benefits of digital technology have been recognized by many companies, researchers have also found that the level of digitalization in traditional manufacturing is still lower than expected [20]. Besides, Luo et al. [21] found that as economic openness, industrial structure, and market potential advance, the promotion intensity of digital economy on green innovation is becoming lower and lower. Therefore, it is necessary to deeply explore the transformation mechanism of the manufacturing industry and further promote the green transformation efficiency of enterprises [22].

To sum up, major research studies focus on digital economy on the manufacturing transformation and upgrading, green economy, industrial economy total factor productivity etc, and more emphasis on industrial green transformation[1, 7, 15]. However, the manufacturing is just a part of industry, and the two cannot be equivalent at all. few studies emphasize the influence of economy on green transformation of manufacturing. and in addition, scholars often use entropy method(EM) and principal component analysis(PCA) to measure the level of green transformation, but it is difficult to fully consider the level of green indicators. Therefore, the Super-SBM model is applied to measure the efficiency of green transformation. To supplement previous research, this study aims to explore this issue in several aspects. Firstly, an improvement is required for the problem of measuring green transformation in the manufacturing industry. To begin with, the Super-SBM model of non-expected output and constrains on industrial waste emissions are taken as non-expected output to calculate the total factor productivity of green transformation in the manufacturing industry. Then, a fixed-effect regression model and a threshold model are applied to account for the non-linear relationship between the digital economy and green transformation in this industry. Furthermore, an intermediate effect model is adopted to validate the internal mechanisms and the influencing paths between the digital economy and green transformation in this industry. The remainder of this paper is structured as follows. In Section 2, the research hypotheses are presented. In Section 3, model selection and data sources are explained in detail. Section 4 shows the analysis results. In Section 5, a discussion is conducted. In Section 6, the paper is concluded with policy recommendations.

## Research hypotheses

### The digital economy can effectively promote the green transformation of the manufacturing industry

Through its cluster breakthroughs in modern information technology and its integration with the manufacturing industry, the digital economy provides a crucial means to digitize and intelligentize the manufacturing industry, which is an important means to reduce resource consumption and beneficial to its green transformation [23]. Using the wine industry as an example, Silvestri et al. [24] found that the implementation of digital technologies facilitates the integration and reallocation of various resources within a company, thus helping it to establish a competitive advantage in the marketplace. Currently, there are three ways in which the digital economy promotes the green transformation of the manufacturing industry. The first one is to empower product lifecycle management and footprint tracking analysis. For manufacturing enterprises, they can develop a clear process of emission reduction and provide technical support for their green development strategies by establishing a basic database for the entire lifecycle of product design, manufacturing, transportation, and sales, and utilizing big data and IoT analysis technologies to optimize the processes and techniques throughout the entire lifecycle. The second one is to reduce energy and material consumption by empowering production process control. Through AI, industrial robots, and other technologies, it is achievable to optimize material scheduling, production equipment operation, and human-machine interaction. Also, the production process can be further optimized and controlled to guide the effective allocation of various resources, improve the rate of equipment utilization, reduce production costs, and ultimately achieve the objective of reducing energy and material consumption. The last one is to empower the recycling and utilization of supply chain resource [22]. With IoT and big data technologies applied to build the platforms of waste resource information service and public technology service, the manufacturing industry can get connected effectively with the information technology service industries to achieve the rapid promotion, matching, connection, and transaction of waste resource trading information, as required to further promote intelligent and coordinated green development. Bhatti et al. [25] found that business model innovation in IT firms was established as a mediator in the relationship between these factors and business performance. In contrast, Niehoff [26] found that industrial digitalization has not only positive but also possible negative effects on corporate sustainability, such as increased resource consumption and larger upfront investments. In the manufacturing industry, the empowerment of green transformation by digital economy shows the advantages of precision, timeliness, and full-process systematization. For acceleration of the comprehensive balanced development of digital industrialization and industrial digitization, there is a need to give full play to the potential of the digital economy in empowering green transformation in the manufacturing industry. As a whole, the digital economy is considered to have a positive impact on the green transformation of industry. On this basis, the following hypothesis is proposed:

H1: The digital economy can effectively promote the green transformation of the manufacturing industry.

### Non-linear spillover benefits of the digital economy on the green transformation of the manufacturing industry

As a novel form of economy, the digital economy plays a significant role in the green transformation of the manufacturing industry. However, its promoting effect is affected by the level of scale and the quality of development [22]. Firstly, the level of scale is low for the digital economy. In the early stages of digital economy development, the scale is relatively low, which is compounded by high investment and long recovery periods. For this reason, most enterprises are unwilling to make investment in new digital technologies. Only those manufacturing enterprises with strong technological capabilities and sufficient funds are willing to try. As the infrastructure of digital economy improves continuously and user scale increases, the marginal cost of the digital economy shows a decreasing trend. In this context, manufacturing enterprises are further encouraged to increase their capital investment in digital technology, deepening into such fields as R&D design, production manufacturing, and waste recycling. Thus, the development of manufacturing is promoted towards green, intelligent, and digitization. At that time, a critical point will be gradually reached by the driving force of the digital economy on the green transformation of manufacturing. Also, the scale of the digital economy is excessively expanded. In the process of integrating the digital economy and the real economy, it is easy for enterprises to break through the traditional scale limits. In some cases, the excessive pursuit of benefits may cause deviation from the purpose of serving the real economy, which presents the risk of "separation from reality and turning to virtuality." Due to the excessive expansion of the digital economy, there is a significant reduction in the amount of capital originally directed by enterprises to improving industrial value, which may lead to the mismatch of production factors. Ultimately, the green transformation of the manufacturing industry is adversely affected. Meanwhile, Hao et al. [27] found that there exists obvious regional spatial heterogeneity and threshold effect between digitalization and green economy growth. So does the digital economy also have such an impact on the green transformation of manufacturing. Therefore, the following hypothesis is proposed:

H2: The digital economy has non-linear spillover benefits on the green transformation of the manufacturing industry.

### The digital economy promotes the green transformation of manufacturing through innovative green technologies and industrial structural upgrading

The capability of green technology innovation provides a significant driving force for the green transformation of the manufacturing industry. Ning et al. [28] using data covering 1,166 listed manufacturing firms in China verify that enterprise digitalization can positively affect green innovation. Scuotto et al. [29] found that intra-organizational (in-house research and development [R&D]) and inter-organizational (open innovation) processes improve SMEs’(small and medium-sized enterprises) innovation performance.As one of the most innovative forms of economy, the digital economy exerts a positive effect on regional innovation through its development. Through the deep integration of the digital economy and manufacturing, the innovation capability of the manufacturing industry can be effectively enhanced and the upgrading of its development momentum can be achieved. Firstly, the digital economy supports enterprises in their shift to open innovation. The key to open innovation is to overcome the constraints of boundaries between companies and other related organizations, and to acquire more innovation knowledge from external sources. Because of the virtual and external nature of the digital economy, the transformation of enterprises towards open innovation is promoted [30]. Secondly, the digital economy is beneficial for enterprises to improve innovation efficiency. As a general technology, digital technology is not exclusive to individual companies anymore, which lowers the innovation threshold for small and medium-sized enterprises. By forming technology-sharing alliances, collaborative research and development among industry, academia and research institutes, and co-building collaborative innovation network platforms, guidance can be provided on the development of energy-saving and emission-reducing technologies and low-carbon technologies. This is essential for achieving green and low-carbon production in the manufacturing industry and steering the industry away from the high-polluting and high-energy-consuming development model. Thirdly, digital technology innovation contributes to increasing added value for the manufacturing industry. Widely used in product development, processing and manufacturing, and information processing in the manufacturing industry, digital technology is effective in improving the efficiency of enterprises in resource allocation and production. Also, it is applicable to transform the manufacturing industry from a low value-added industry that is resource and labor-intensive to a high value-added industry that is technology and knowledge-intensive. Therefore, the following hypothesis is proposed:

H3a: The digital economy promotes green transformation in manufacturing through green innovation.

In essence, industrial upgrading is purposed to enhance the efficiency in allocating various production factors between different industries, and to improve the industrial structure by strengthening the effective integration among different industries. In the view of Hu Shupeng [31], the industrial structure is made more reasonable by industrial structure upgrading, which reduces the unit energy consumption, and which enhances the efficiency of energy utilization significantly, and improves the level of green manufacturing effectively. Jafari and Rezaee [32] argue that the upgrading of industries will improve the coordination and resource utilization among industries and further contribute to the green transformation. Firstly, the increase in data factor input not only further enriches the dimension of transformation between factors and products, but also promotes the transfer of production factors from low-efficiency industries to high-efficiency industries, thus improving the overall utilization efficiency of various production factors and promoting industrial structure upgrading. Secondly, the boundaries of technology are broadened by the widespread application of new-generation digital information technologies, with artificial intelligence and blockchain as the representatives. This gives rise to new forms of industries, such as new production models, new retail models, and new industries, thus promoting the in-depth integration among different industries and driving industrial transformation and upgrading. Finally, the development of the digital economy relies on a large number of comprehensive high-tech and high-knowledge talents, and the improvement of human capital level causes a shift in the structure of industrial employment, which promotes industrial structure upgrading. Due to the introduction of data as a special production factor, the structure of factor allocation in various industries is optimized, which not only provides a further guidance on industrial structure upgrading but also improves the level of green development in the manufacturing industry. Therefore, the following hypothesis is proposed:

H3b: The digital economy drives green transformation in manufacturing through promoting industrial structure upgrading.

## Methods, variable selection, and data sources

### Methods

Firstly, the digital economy is factored into the analysis framework of the green transformation in the manufacturing industry. Thus, a basic dynamic panel regression model is constructed as follows:

$$MT_{it}=\alpha +\alpha_{1}DE_{it}+\alpha_{c}•X_{it}+\chi_{i}+\eta_{i}+\varepsilon_{it}$$ (3-1)

where, *MT*_*it*_ represents the level of green transformation in the manufacturing industry of province *i* in period *t*; *DE*_*it*_ refers to the development index of digital economy in province *i* in period *t*; *X*_*it*_ represents five control variables including economic development level, trade openness, foreign direct investment intensity, environmental regulation, and policy support in province *i* in period *t*; *α*_*c*_ denotes the estimation parameter; *λ*_*i*_ and *η*_*i*_ represent the individual fixed effects of province *i* that do not change over time and the time fixed effects, respectively; α indicates the fixed intercept; and *ε*_*it*_ denotes the random error term.

Furthermore, a test is conducted to verify whether there is a nonlinear relationship between the digital economy and the green transformation in the manufacturing industry. Based on Model 3–1, a panel threshold model is constructed as follows:

$$MT_{it}=\alpha +\alpha_{0}MT_{it-1}+\alpha_{1}DE_{it}*I(DE_{it}\leq \gamma^{\prime })+\alpha_{2}DE_{it}*I(DE_{it}\geq \gamma^{\prime })+\alpha_{c}•X_{it}+\chi_{i}+\eta_{i}+\varepsilon_{it}$$ (3-2)

*DE*_*it*_ is not only the core explanatory variable of the model, but also a threshold variable. *γ*′ represents the threshold value to be estimated, with *I*(•) as an indicator function. Depending on the number of threshold values, there can be multiple cases. In practice, the model is tested using Stata’s maximum threshold value of 3, through which it can be determined how many thresholds exist in the model.

Furthermore, the mechanisms behind the green transformation of the digital economy and manufacturing industry are examined in the study through an investigation into whether three mediator variables play a significant role, namely green technological innovation capability, industrial transformation and upgrading, and industrial transformation rationalization. The stepwise regression method proposed by Wen Zhonglin et al. [33] is used to construct a specific mediation effect model.


$$W_{it}=\beta +\beta_{1}DE_{it}+\beta_{c}•X_{it}+\chi_{i}+\eta_{i}+\varepsilon_{it}$$ (3-3)

$$MT_{it}=\gamma +\gamma_{1}DE_{it}+\gamma_{2}W_{it}+\gamma_{c}•X_{it}+\chi_{i}+\eta_{i}+\varepsilon_{it}$$ (3-4)

### Variable selection

#### Level of green transformation in the manufacturing industry

As for the explanatory variable for the level of green transformation in the manufacturing industry, it is measured by the efficiency of green transformation development in the manufacturing industry, with both economic and environmental performances taken into account. Based on the availability of data on the provincial level manufacturing sector, the labor input in the manufacturing industry is used to measure input variables. Among them, capital input is represented by the total fixed assets investment of the above-scale manufacturing industry, while energy input is referred to as the electricity consumption of the manufacturing industry [34].

In terms of output, expected output is measured against the total output value of above-scale manufacturing enterprises, while unexpected output represents the negative impact caused by the manufacturing process on the environment. Following the approach of Khan et al. [35], unexpected output is represented in the study by three industrial wastes (industrial wastewater discharge, industrial sulfur dioxide emissions, and industrial solid waste generation), as shown in Table 1. The Super-SBM model, which gives consideration to unexpected output, is applied in this study to measure the level of green transformation in the manufacturing industry.

**Table 1:** Relevant indicators for green transformation in the manufacturing industry.

**Explanatory variables: Manufacturing Green Transformation**	Input indicators	Number of employees in manufacturing industry (person)
		Manufacturing fixed asset investment (100,000,000 yuan)
		Manufacturing Industry Electricity (Coal) (10,000 tons)
	Output indicators	Manufacturing output value above designated size (100,000,000 yuan)
	Undesired output	Industrial waste (100,000,000 tons)
**Explained variable**		Digital Economy
**Mediator variable**		Green Technology Innovation Capability
		Industrial structure upgrade
**Control variable**		Level of Economic Development
		Trade Openness
		Intensity of foreign direct investment
		Environmental Regulation
		Policy Support

#### Level of development in the digital economy

The dependent variable used in this study is the level of development in the digital economy. According to scholars’ interpretations of the concept and connotation of the digital economy, an evaluation index system is constructed in this study by using four dimensions and 22 tertiary indicators, such as digital economic development carriers, digital industrialization, industrial digitization, and the development environment of the digital economy [36]. The entropy weighting method is used to perform measurement. Also, the data is first standardized before the calculation of information entropy, weight, and comprehensive scores, so as to avoid the estimation errors caused by dimensional differences between different categorical variables.

### Control and mediator variables

A number of suitable control variables are selected to accurately evaluate the impact of the digital economy on the level of green transformation in the manufacturing industry and to minimize the interference caused by other factors. By referring to scholars such as Emrouznejad and Yang [11] and Liu Xinzhi and Kong Fangxia [12], the following control variables are selected: economic development level, which is represented by per capita GDP; degree of trade openness, which is indicated by the ratio of total imports and exports to GDP; degree of foreign direct investment, which is referred to as the ratio of net fixed assets investment of foreign investment to total net fixed assets investment; environmental regulation, which is usually represented by the comprehensive utilization rate of industrial solid waste as a proxy variable. Playing a vital role in promoting the green transformation of the manufacturing industry, government policy support is represented by the proportion of general government budget support to regional GDP.

Finally, green technological innovation capability and industrial structural upgrading are taken as mediator variables, which is based on the research hypothesis. As for the indicator for green technological innovation capability, it is measured against the number of new product development projects in the manufacturing industry. Industrial structural upgrading is reflected mainly in the rationalization (*Itu*_*R*_) and advancement (*Itu*_*A*_) of industrial transformation and upgrading. The rationalization of industrial structure reflects not only the degree of industry agglomeration quality and coordinated development, but also the effective utilization rate of various production factors. Industrial structural advancement is used to describe the iterative feature of industrial structure shifting from a low level to a high level [37]. Referring to Brandt [38], Fan and Li [39], this study uses industrial structural upgrading as a mediating variable and represents industrial structural upgrading in terms of rationalization and advancement of industrial transformation and upgrading. Specifically, industrial structure rationalization is measured by introducing the importance of industry into the deviation measurement formula, It is redefined as:

$$Itu_{R}=\sum_{i=1}^{n}\left(\frac{Y_{i}}{Y}\right)\ln(\frac{\frac{Y_{i}}{L_{i}}}{\frac{Y}{L}})$$ (3-5)

where, *Y* represents the output value of a certain type of industry, and *L* refers to the number of employees in a certain type of industry. In this study, they are divided into the first, second, and third industries according to the national economic accounting standards. *Itu*_*R*_ refers to the equilibrium state of the economy. When the *Itu*_*R*_ value decreases, it indicates a more reasonable economic structure in the industry. Conversely, if the value increases, it suggests an unbalanced economic state and an unreasonable structure. The advancement of industrial transformation and upgrading is reflected in this study by the proportion of the output value of the third industry to the output value of the second industry.

### Data source and descriptive statistics

Allowing for the accuracy and availability of data, 31 provinces in China (excluding Hong Kong, Macao, and Taiwan) are selected in this study as the sample, covering the period from 2011 to 2020. The data used for this study is sourced mainly from the "China Statistical Yearbook", "China Information Statistical Yearbook", "China Fixed Asset Investment Statistical Yearbook", "White Paper on the Development of the Digital Economy", and others. As for the missing data in the sample, a variety of methods are typically used for imputation, such as analogy, interpolation, and proportional weighting. Table 2 shows the descriptive statistical results of the four types of variables used in this chapter.

**Table 2:** Descriptive statistics of variables.

variable	N	mean	p50	sd	min	max
**MT**	310	0.171	0.116	0.187	0.00600	1.230
**DE**	310	0.204	0.146	0.201	0.0200	0.955
**Pgdp**	310	53484	45753	27548	16165	164889
**Open**	310	201.7	0.163	605.4	0.00700	2015
**Fdi**	310	1146	610.2	1583	2.700	8587
**Evr**	310	0.583	0.601	0.290	0	0.998
**PS**	310	0.297	0.238	0.210	0.119	1.354
**Gic**	310	14639	7453	23471	7	166140
***Itu***_***R***_	310	1.225	1.073	0.686	0.518	5.297
***Itu***_***A***_	310	0.210	0.190	0.134	0.00800	0.651

Abbreviations: MT(Manufacturing Green Transformation); DE(Digital Economy); Pgdp(Level of Economic Development); Open(Trade Openness); Fdi(Intensity of foreign direct investment); Evr(Environmental Regulation);PS(Policy Support);Gic(Green Technology Innovation Capability); *Itu*_*R*_ (Industrial structural upgrading is reflected mainly in the rationalization); *Itu*_*A*_ (Industrial structural upgrading is reflected mainly in the advancement).

## Empirical analysis

### Regression results of the baseline model

Prior to the baseline regression analysis, it is necessary to determine whether the sample data is suitable for regression analysis. Besides, some variables are logged before correlation analysis to avoid the estimation bias caused by dimensional differences between different categorical variables, with the results shown in Table 3 below. It can be found out that there is a significant correlation between each two variables, except for the degree of trade openness. To verify whether the correlation results from multicollinearity, a collinearity diagnosis is conducted, with the results shown in Table 4 below. The VIF values are all less than 10, indicating that no serious collinearity problem arises and regression analysis can be conducted.

**Table 3:** Correlation analysis.

MT	DE	Pgdp	Open	Fdi	Evr	PS	
**MT**	1						
**DE**	0.948***	1					
**Pgdp**	0.502***	0.594***	1				
**Open**	0.0360	0.0140	-0.0340	1			
**Fdi**	0.692***	0.693***	0.592***	0.0100	1		
**Evr**	0.338***	0.395***	0.498***	0.0890	0.463***	1	
**PS**	-0.430***	-0.447***	-0.357***	0.0260	-0.786***	-0.416***	1

* *p* < 0.1

** *p* < 0.05

*** *p* < 0.01

**Table 4:** Collinearity analysis.

Variable	VIF	1/VIF
**Fdi**	4.760	0.210
**PS**	2.940	0.340
**DE**	2.200	0.454
**Pgdp**	1.980	0.506
**Evr**	1.480	0.673
**Open**	1.020	0.976
**Mean**	VIF	2.400

* *p* < 0.1

** *p* < 0.05

*** *p* < 0.01

Then, model comparison and selection are performed through Hausman test and likelihood ratio (LR). The test results are listed in Table 5 below. As shown in the table, the *p*-value is smaller than 0.01, which rejects the null hypothesis of rejecting the mixed effects model (OLS) and random effects model (RE) being superior to the fixed effects model (FE). Therefore, the fixed effects model is used in this study for regression analysis estimation. Model F (4) and Model F (5) are the results of the analysis without and with all control variables included, respectively. Both models pass the significance test, indicating that the overall explanatory power of the model is strong. According to the analytical results, the 1% significance test is passed by the estimated coefficient of the level of digital economic development on the green and low-carbon transformation of the manufacturing industry after the control variables are included. That is to say, the development of the digital economy plays a significant role in promoting the green transformation of the manufacturing industry, which verifies Hypothesis 1.

**Table 5:** Model estimation results.

	F(1)	F(2)	F(3)	F (4)	F (5)
	**OLS**	**FE**	**RE**	**MT**	**MT**
**DE**	0.866***	0.249***	0.816***	0.880***	0.866***
	(0.023)	(0.092)	(0.040)	(0.017)	(0.023)
**Pgdp**	0.053***	0.062***	0.061***		0.053***
	(0.010)	(0.011)	(0.011)		(0.010)
**Open**	-0.000	-0.000***	-0.000*		-0.000
	(0.000)	(0.000)	(0.000)		(0.000)
**Fdi**	0.025***	0.008	0.021***		0.025***
	(0.005)	(0.007)	(0.006)		(0.005)
**Evr**	-0.016	-0.006	-0.041***		0.016
	(0.013)	(0.012)	(0.011)		(0.013)
**PS**	0.079***	-0.250**	-0.005		0.079**
	(0.026)	(0.099)	(0.042)		(0.026)
**_cons**	0.392***	0.821***	0.557***		0.392***
	(0.097)	(0.119)	(0.113)		(0.097)
**N**	310.000	310.000	310.000		310
**r2**	0.914	0.170			0.912
**r2_a**	0.912	0.061			
		Prob>chi2 = 3.41e-09			

Standard errors in parentheses

* *p* < 0.1

** *p* < 0.05

*** *p* < 0.01

### Robustness analysis model

In this section, further robustness tests are conducted to verify the accuracy of the research findings, despite the benchmark regression analysis showing that the development of the digital economy promotes the green transformation of manufacturing to a significant extent. The robustness analysis results are listed in Table 6 below.

**Table 6:** Results of robustness analysis tests.

	F(6)	F(2)	F(8)	F(9)	F(10)	F(11)	F(12)
	**MT(finance)**	**MT**	**MT(lag term)**	**MT**	**MT**	**MT**	**MT**
**DE**	0.298***	0.249***	0.799***	0.932***	0.931***	0.877***	0.878***
	(0.102)	(0.092)	(0.164)	(0.020)	(0.020)	(0.023)	(0.023)
**Pgdp**	0.122***	0.062***	0.060***	0.039***	0.039***	0.051***	0.046***
	(0.042)	(0.011)	(0.017)	(0.009)	(0.009)	(0.009)	(0.010)
**Open**	0.000***	0.000***	0.000**		0.000**	0.000**	0.000**
	(0.000)	(0.000)	(0.000)		(0.000)	(0.000)	(0.000)
**Fdi**	0.005	0.008	0.002			0.014***	0.015***
	(0.007)	(0.007)	(0.008)			(0.003)	(0.003)
**Evr**	0.026**	-0.006	0.001				-0.024
	(0.011)	(0.012)	(0.021)				(0.013)
**PS**	-0.009	-0.250**					
	(0.111)	(0.099)					
**L2.DE**			-0.240**				
			(0.098)				
**_cons**	-1.095**	0.821***	0.690***	0.404***	0.397***	0.462***	0.411***
	(0.452)	(0.119)	(0.185)	(0.096)	(0.096)	(0.095)	(0.098)
**N**	310.000	310.000	248.000	310	310	310	310
**r2**	0.204	0.170	0.240	0.904	0.904	0.909	0.910
**r2_a**	0.099	0.061	0.111				

Standard errors in parentheses

* *p* < 0.1

** *p* < 0.05

*** *p* < 0.01

Firstly, by referring to the research conducted by Wang et al. [40], the "Peking University Digital Finance Inclusion Index" (https://idf.pku.edu.cn/yjcg/zsbg/index.htm) is taken as a proxy variable in the robustness test to measure the level of digital economic development. According to the analytical result F(6), the coefficient of the core explanatory variable is consistent with the F(5) result. The Digital Finance Inclusion Index shows a positive correlation with the green transformation of manufacturing, passing the 1% significance test, indicating the robustness of the result.

Secondly, this research relieson two-stage least squares (2SLS) and two-stage difference generalized method of moments (DIFF-GMM) to carry out estimation and test, respectively. Apart from that, the lagged digital economic development index is introduced as an instrument variable to apply control on potential endogeneity, with the model extended to a dynamic panel data model for dynamic estimation. According to the F(8) result, the lagged digital economic development level continues to exert a positive effect on the green transformation of manufacturing, passing the 5% significance test, which indicates the robustness of the result.

Thirdly, the robustness test is conducted by gradually introducing control variables to determine whether there is endogeneity caused by either omitted variables or bidirectional causality in the fixed-effect model of the basic regression. Given different combinations, the regression coefficients pass the 5% significance test in the F(9), F(10), F(11), and F(12) models, which confirms a significant and stable positive impact caused by the development of the digital economy on the green transformation of manufacturing.

### Threshold effect analysis

In this study, the impact of digital economy development on green transformation of the manufacturing industry is accurately described by conducting a threshold effect analysis with the green transformation level of the manufacturing industry as the dependent variable and the digital economy development index as the threshold variable. Prior to the threshold analysis, it is necessary to determine whether there is a threshold effect between the variables. According to the test results shown in Table 7, the test statistics are 41.1, 26.3, and 15.1 for the single threshold, double threshold, and triple threshold of the digital economy development level, respectively. It is indicated that the triple threshold fails the significance test. Therefore, the number of thresholds is set to 2, while the estimated values are 0.144 and 0.283 for the single threshold and double threshold, respectively.

**Table 7:** Test results of threshold effects between the variables.

Category	Single threshold	Double sill	Three thresholds
**F-statistic**	41.4	26.3	15.1
**P value**	0.005	0.0067	0.203
**Threshold 1%**	37.92	13.810	17.661
**Threshold 5%**	32.98	16.937	20.996
**Threshold 10%**	29.34	24.100	33.404
**threshold estimate**	0.1440	0.283	0.324
**95% confidence interval**	[0.133,0.146]	[0.279,0.299]	

As shown in Table 8, when the digital economy development level falls below 0.144, the coefficient of the impact of digital economy development on green transformation of the manufacturing industry is positive but insignificant. When the digital economy development level ranges between 0.144 and 0.283, the estimated coefficient passes the significance test at 10%. When the digital economy development level exceeds 0.283, the estimated coefficient is 0.399, passing the significance test at 5%. That is to say, as the level of digital economy development increases, the green transformation of the manufacturing industry is promoted more effectively. As a result, there is a non-linear relationship with an increasing marginal effect that varies with the threshold variable of digital economy development. Thus, Hypothesis 2 is verified.

**Table 8:** Estimation results of threshold effects.

Variable	Coefficient	T value	Standard error
**Pgdp**	0.067***	4.871	0.017
**Open**	0.000***	4.570	0.000
**Fdi**	0.007	1.043	0.006
**Evr**	-0.008	-0.158	0.011
**PS**	-0.269***	-2.980	0.091
**DE<0.144**	0.133	-2.870	0.194
**0.144≤DE <0.283**	0.237*	1.562	0.118
**0.283≤DE**	0.399**	1.887	0.173
**_cons**	0.875***	6.330	0.148

* *p* < 0.1

** *p* < 0.05

*** *p* < 0.01

### Mediation analysis

As shown in Table 9, models F(13), F(14), and F(15) are used to test the mediation effects of technological innovation capability, industrial transformation and upgrading to advanced levels, and industrial rationalization, respectively. Firstly, in terms of technological innovation capability, the estimate coefficient for digital economic development is 0.249, passing a 1% significance test in the first column. By comparison, the estimate coefficient for digital economic development is 1.158 in the second column, passing a 5% significance test. It means that digital economy contributes to improving the capability of technological innovation under other conditions. In the third column, the impact coefficient of digital economic development on manufacturing green transformation declines from 0.249 to 0.211, passing a 5% significance test. It demonstrates a mediating role played by the improvement in technological innovation capability. Secondly, in terms of industrial transformation and upgrading to advanced levels, the F(14) result shows no statistically significant relationship of industrial transformation and upgrading to advanced levels with manufacturing green transformation. Finally, in terms of industrial rationalization, industrial rationalization exerts partial mediation effects on digital economic development and manufacturing green transformation. In the second column, the estimate coefficient for digital economic development is -0.176, passing a 1% significance test, indicating the promoting effect of digital economy on the improvement of industrial rationalization. In the third column, there is a decrease from 0.249 to 0.214 in the impact coefficient of digital economic development on manufacturing green transformation, which passes a 5% significance test, demonstrating a mediating role played by the improvement in industrial rationalization.

**Table 9:** Mediation analysis results.

	F (13)			F (14)		F (15)	
	**MT**	**Gic**	**MT**	***Itu***_***A***_	**MT**	***Itu***_***R***_	**MT**
**Pgdp**	0.249***	1.158**	0.211**	0.367***	0.233**	-0.176***	0.214***
	(0.093)	(0.536)	(0.092)	(0.491)	(0.092)	(0.107)	(0.093)
**Open**	0.063***	1.143***	0.096***	0.991***	0.031**	0.202***	0.082***
	(0.011)	(0.062)	(0.016)	(0.057)	(0.015)	(0.012)	(0.015)
**Fdi**	0.000**	-0.000***	0.000***	0.000	0.000**	-0.000	0.000**
	(0.000)	(0.000)	(0.000)	(0.000)	(0.000)	(0.000)	(0.000)
**Evr**	0.009	0.026	0.008	-0.154***	0.004	0.007	0.010
	(0.007)	(0.043)	(0.007)	(0.039)	(0.008)	(0.009)	(0.007)
**PS**	-0.009	-0.069	-0.007	0.025	-0.009	0.009	-0.009
	(0.012)	(0.068)	(0.012)	(0.063)	(0.012)	(0.014)	(0.012)
**Mediator variable**			0.229***		0.033		0.294**
			(0.010)		(0.011)		(0.052)
**Mediating effect**		Presence		No Presence		Presence	
**Mediating effect percentage**		21.2%				21.8%	
**N**	310.000	310.000	310.000	310.000	310.000	310.000	310.000

Standard errors in parentheses

* *p* < 0.1

** *p* < 0.05

*** *p* < 0.01

## Discussion

In the manufacturing industry, there are some outstanding problems such as the vulnerabilities in innovation, high energy consumption, and severe environmental pollution, despite the world’s largest and most extensive manufacturing system that have been established. Data, as a new production factor, represents a new type of industrial productivity that can promote the green transformation and upgrading of manufacturing industry. Therefore, it is significant to explore not only the relationship between the digital economy and the green transformation of the manufacturing industry but also the underlying mechanism of it. According to the results of our empirical analysis, the digital economy has a significant positive impact on the green transformation of the manufacturing industry, which verifies Hypothesis 1. As for the promoting effect of the digital economy on the green transformation of the manufacturing industry, it is affected by the size of the threshold variable, showing a non-linear relationship of "marginal effect" increasing, which verifies Hypothesis 2. According to the results of the mediation analysis, the capability of technological innovation and the rationalization of industrial transformation and upgrading have significant partial mediation effects, while the high-level industrial transformation and upgrading exert no mediation effect, which means Hypothesis 3 is not fully validated.

### The relationship between digital economy and green transformation of manufacturing

Our empirical results reveal that digital economy exerts a significant promoting effect on the green transformation of the manufacturing industry, which is consistent with the conclusions drawn in the research of Kong Fangxia and Liu Xinzhi [12], and Wurlod et al. [41]. However, compared to previous research results, there are significantly differences in the analytical results after the introduction of all control variables [21]. The results of F(5) analysis show that the estimated coefficient of economic development level on the green transformation of the manufacturing industry is 0.053, reaching a significant extent. That is to say, the improvement of regional economic development level contributes to the green transformation of the manufacturing industry. This may be because the better the economic development, the more people pay attention to environmental issues, which prompts the government to strengthen environmental regulation and increase the investment in green innovation funds, thus promoting the green transformation of the manufacturing industry. The estimated coefficient of the degree of trade openness on the green transformation of the manufacturing industry is negative but insignificant, indicating that the current degree of trade openness exerts no positive impact on the green transformation of the manufacturing industry. In China, the development of low-tech labor-intensive processing trade has not been completely steered away from the expansion mode of low value-added, and the green transformation of the manufacturing industry is hindered by the export trade with low-price competitive advantages. The estimated coefficient of foreign direct investment level on the green transformation of the manufacturing industry is 0.025, which is significant at the 1% level. It is indicated that foreign direct investment can improve the level of green transformation in the manufacturing industry by bringing new production technology, research and development capabilities, management concepts, and other factors. The estimated coefficient of environmental regulation on the green transformation of the manufacturing industry is positive but insignificant. Due to the increase in intensity of environmental regulation, manufacturing companies are forced into adopting clean technology and reducing pollutant emissions. However, there is no significant positive effect exerted on the green transformation of the manufacturing industry due to the lack of legislative constraints and weak enforcement in the Chinese environmental regulation system. The estimated coefficient of policy support on the green transformation of the manufacturing industry is positive and significant at the 5% level. That is to say, a driving force of green innovation in the manufacturing industry is provided by the government’s support in terms of technological upgrading, fund assistance, tax reductions, and other areas, which promotes the green transformation of the manufacturing industry.

There is a non-linear relationship exhibited by the impact of the digital economy on the green transformation of the manufacturing industry. Also, its promoting effect becomes more significant with the increase in the level of digital economic development, which is basically coherent with Okaily’s findings [17]. However, there are significant differences between the two studies in the measurement of the level of digital economy and the level of green transformation of the manufacturing industry, which results in different threshold values and coefficients for the threshold variable, that is, the level of digital economic development. Specifically, there are variations by the level of scale and quality in the effect of digital economic development on the green transformation of the manufacturing industry. Additionally, this study introduces a new factor, unexpected output, with the Super-SBM model used to measure the efficiency of green transformation in the manufacturing industry for each province. Thus, the effect of green transformation in each province can be reflected in a more intuitive and accurate way.

### The mediating effect of the digital economy and the green transformation of manufacturing

There is a complex process involved in the mechanism by which the digital economy promotes the green transformation of manufacturing. To reveal how the digital economy affects green transformation of manufacturing, we consider the rationalization and advancement of industrial transformation and upgrading, and innovation capability of green technology as the mediator and explore its impact. We find that rationalization of industrial transformation and upgrading, innovation capability of green technology contributes to the green transformation of manufacturing, but the advancement of industrial transformation and upgrading does not show a mediating effect.

Firstly, with regard to the advanced upgrading of the industry, the F(14) results indicate no significant statistical relationship between the advanced upgrading of the industry and the green transformation of the manufacturing industry, which contradicts Hypothesis 3, which is consistent with prior studies [39]. The possible reasons for this are as follows. On the one hand, information technology and the service industry, as represented by big data and artificial intelligence, are inherently complementary to each other, which causes the digitization of the industry to be concentrated in the service industry. During the process of industrial transformation and upgrading, there are more data elements flowing into the service sector of the tertiary industry but less to the manufacturing sector of the secondary industry. On the other hand, the traditional manufacturing industry shows tangible economic attributes, and it is a relatively difficult, costly, and time-consuming process to apply digital technology in the manufacturing industry, which further constrains the manufacturing industry from green transformation.

Secondly, with regard to the rationalization of industrial transformation and upgrading, there is a partial mediating effect exerted on the development of the digital economy and the green transformation of the manufacturing industry. This is contrary to the conclusions drawn in the research of Kong Fangxia and Liu Xinzhi [12], and Huining and Yangxin [42]. This may be because the digital economy infrastructure is relatively well-developed in China, and the government has also created a favorable internal and external environment for the development of the digital economy by introducing various preferential support policies. By strengthening the cooperation between different types of enterprises, the rational allocation of various factors is promoted, such as human resources, capital, and technology, while the cooperation level of factor endowments is improved. It leads to the further rationalization of employment, personnel and industrial structures, thus promoting the low-carbonization of energy consumption, the greening of product supply, and the circular utilization of resources. Ultimately, the green transformation of the manufacturing industry is promoted.

Thirdly, through comparison of the results, it can be found out that the impact of industrial upgrading rationalization on the green transformation of the manufacturing industry is more significant compared to industrial upgrading advanced upgrading. Therefore, when industrial restructuring is conducted, it is essential to take a rational view on industrial transformation and upgrading, pay more attention to the aggregation quality of industrial structures and the coordination among various factors, and promote the rationalization of industrial structural transformation for the better green transformation of the manufacturing industry.

There are several limitations to this study. Firstly, this study takes into account only industrial waste as adverse emissions. However, there are many other types of greenhouse gases emitted in the manufacturing process, such as carbon dioxide and methane. Therefore, it is necessary to comprehensively consider the emissions of greenhouse gases such as carbon dioxide for a more accurate evaluation of the green transformation efficiency in the manufacturing industry. Secondly, there are many driving paths between the digital economy and the green transformation of the manufacturing industry. In this study, the mediating effects of green technology innovation capability, advanced upgrading, and rationalization of industrial transformation and upgrading are examined, but they are not the only pathways. In the future, a comprehensive consideration will be given from the enterprise level to such factors as financing capability, human capital, and R&D investment intensity for the further exploration into the path by which the digital economy promotes the green transformation of the manufacturing industry.

## Conclusion and policy suggestions

### Conclusion

There is an opportunity presented by the deep integration of the digital economy and the manufacturing industry for the green transformation of the manufacturing industry. In this study, econometric models are used to explore the relationship between the digital economy and the green transformation of the manufacturing industry for analysis of the relevant mechanisms from different perspectives including green technology innovation, advanced upgrading, and rationalization of industrial transformation and upgrading. On this basis, the following conclusions are drawn. Firstly, the digital economy exerts a significant promoting effect on the green transformation of the manufacturing industry, which passes various robustness tests. Secondly, the significant threshold of the development level of the digital economy is a constraint on the efficiency of green transformation in the manufacturing industry. Within different ranges of threshold value, there are variations in the impact coefficient of the digital economy on the green transformation of the manufacturing industry, showing a non-linear characteristic of "marginal effect" increasing. Thirdly, green technology innovation provides an important means for the digital economy to drive the green transformation of the manufacturing industry. According to the results of empirical analysis, the innovation capability of green technology exerts a significant mediating effect on the improvement of the green transformation efficiency of the manufacturing industry through the digital economy. Lastly, despite a significant positive promoting effect of the digital economy on the rationalization and advanced upgrading of industrial structure, the advanced upgrading of industrial structure does not promote the green transformation of the manufacturing industry like the rationalization of industrial structure, nor does it show a partial mediating effect.

The current research work provides a new perspective for studying the green transformation of the manufacturing industry, and also helps to clarify the internal mechanism of the digital economy driving the green transformation of the manufacturing industry, further enriching the relevant theories of green transformation in the manufacturing industry. In practice, the impact mechanism of digital economy on manufacturing transformation and upgrading is empirically tested, which provides a basis for better policy formulation and green transformation of manufacturing industry.

### Policy suggestions

Based on the above conclusions, the following recommendations are made. The first one is to accelerate the development of the digital economy and empower the green transformation of the manufacturing industry by taking advantage of the digital economy. Therefore, importance should be attached to improving the construction of digital infrastructure, consolidating various digital technology foundations such as 5G and big data centers, promoting the development of digital economy aggregation, releasing the dividends of digital economy development to the full, breaking the threshold constraint of the digital economy, improving the efficiency of resource utilization in an all-round way, and supporting the green and digital transformation and upgrading of traditional manufacturing industry. The second one is to accelerate digital innovation and stimulate the vitality of manufacturing enterprises in green technology innovation. For the government, such policies as tax relief and supporting public infrastructure can be exercised to create a favorable environment for activating green technology innovation. From the perspective of manufacturing enterprises, it is necessary to actively explore the new models of digital transformation, apply digital technology in such activities as R&D design, production and operation, energy conservation, and emission reduction, as well as improve the capability of green technology innovation in the manufacturing industry. The last one is to optimize industrial structure for the creation of a new path through which the digital economy can promote the green transformation of the manufacturing industry. It is necessary to make full use of financial and credit support, fiscal policies and other means for the efficient allocation of various industrial resources, promote the rational development of various regional industrial structures, eliminate low-efficiency and low-value-added industries, facilitate the deep integration of the digital economy and the green transformation of the manufacturing industry, and embark on a path of green and low-carbon development.

## Supporting information

S1 File (ZIP)
